# The Relationship Between Acne Vulgaris and Insulin Resistance

**DOI:** 10.7759/cureus.34241

**Published:** 2023-01-26

**Authors:** Nazik H Hasrat, Asaad Q Al-Yassen

**Affiliations:** 1 Family and Community Medicine, College of Medicine, University of Basrah, Basrah, IRQ

**Keywords:** basrah, hyperinsulinemia, tyg index, c-peptide, insulin resistance

## Abstract

Acne vulgaris is a chronic inflammatory disease of the pilosebaceous unit that usually affects adolescents. The aetiology and severity of acne may be influenced by hyperinsulinemia and insulin resistance. A case-control study was conducted in the dermatology outpatient clinic of Al-Fayhaa Teaching Hospital in Basrah city. C-peptide and triglyceride-glucose (TyG) index levels were measured in 43 acne vulgaris patients who were age- and gender-matched with 48 controls. The results found that 81% and 67% of acne patients have insulin resistance based on their C-peptide and TyG index levels, respectively, and this is significantly higher than the control candidates (P = 0.001). Furthermore, a higher value of C-peptide was found among acne cases compared to controls (2.84 ± 1.23 vs. 1.68 ± 0.19) (P = 0.001), and a higher value of the TyG index was found among cases compared to controls (4.43 ± 0.17 vs. 3.38 ± 0.19) (P = 0.001), in addition to a significant positive and strong correlation between the level of these biomarkers and the acne severity (r = 0.760 for the C-peptide and 0.814 for the TyG index) (P = 0.001).

## Introduction

Acne vulgaris is a self-limiting inflammatory condition of the pilosebaceous unit that has a chronic course. Seborrhoea, open and closed comedones, erythematous papules and pustules, and, in more severe cases, nodules, deep pustules, and pseudocysts are all manifestations. Scarring is likely to occur in many cases [1].

Multiple factors have been implicated in the pathogenesis of acne vulgaris. Nonetheless, four major causes have been identified as primary influences on its occurrence: increased sebum production, pilosebaceous duct hypercornification, abnormal microbial flora, particularly Propionibacterium acnes colonization, and inflammation [2].

A relationship between acne and insulin resistance exists; hence, it is found that hyperglycemic carbohydrates and insulinotropic milk and dairy products are associated with diabetes and may promote acne pathogenesis by encouraging insulin growth factor-1 (IGF-1) signaling, which may support the link between milk products and acne [3]. Furthermore, insulin promotes androgen synthesis, resulting in excessive sebum production, a known correlate of acne severity [4]. It has also been demonstrated that a low-glycemic index diet improves acne severity as well as insulin sensitivity in young males with acne vulgaris. As a result, insulin and carbohydrate metabolism may play a role in the cause and severity of acne [5].

As there is a lack of studies to find out whether insulin resistance occurs in acne patients, especially among the Iraqi population, the present study was aimed at determining the association between insulin resistance and the development of acne vulgaris.

## Materials and methods

A case-control study was conducted from January 1, 2022, to April 1, 2022, in the dermatology outpatient clinic of Al-Fayhaa Teaching Hospital in Basrah city. University of Basrah, College of Medicine Research Ethics Committee issued approval 8/39/331. Forty-three patients who fulfilled the inclusion criteria were age- and sex-matched with 48 controls recruited from another outpatient clinic. The inclusion criteria for the study were: patients with acne vulgaris who were willing to participate in the study and agreed to collect blood samples for laboratory testing; while subjects on any medications known to affect insulin metabolism including topical steroids, previous treatment with oral retinoids or any hormonal treatment for any reason in the previous three months, cigarette smoking, a history of diabetes mellitus, hypertension, psoriasis, polycystic ovarian disease, women with menstrual irregularities, known hormonal dysregulations or any other known metabolic disorders were excluded from the study.

The researcher used a specific scoring system for the assessment of severity called the Global Acne Grading System (GAGS). According to this score, acne was graded as mild, moderate, severe, and very severe [6].

A peripheral venous blood sample was collected from all the participants under aseptic conditions after more than eight hours of fasting. C-peptide, fasting blood sugar, and triglyceride level assessments were done for them. Plasma glucose levels and triglyceride levels were measured by the Cobas Integra 400 Plus (Roche, Basel, Switzerland). The triglyceride-glucose (TyG) index was calculated as the natural logarithm (Ln) of the product of plasma glucose and triglyceride (TG) using the formula: Ln (TG [mg/dL] × glucose [mg/dL] /2) [7]. Subjects with an index of 4.49 or greater are likely to suffer from insulin resistance [8]. C-peptide was measured using the CL-1000i Chemiluminescence Immunoassay System (Mindray, Shenzhen, China), and a value higher than 1.89 ng/ml was the cutoff used for the definition of insulin resistance [9].

The dataset was coded and analyzed using the Statistical Package for the Social Sciences (SPSS) version 26 (IBM Corp., Armonk, NY, USA). The numerical data was tabulated as mean and standard deviation (SD). The independent sample student t-test was used to compare the two groups, and ANOVA analysis was used to compare the means of more than three groups. The Chi-square test was used to analyze the qualitative data, which was tallied as a percentage. The Pearson correlation test was used to assess the strength and direction of association between the study variables. A P value of 0.05 or less is considered statistically significant and a P value of 0.01 or less is considered statistically highly significant.

## Results

In this study, 43 cases and 48 controls were included. Female patients made up 34 (79.06%) of the cases and 34 (70.84%) of the controls, while male patients made up nine (20.94%) of the cases and 14 (29.16%) of the controls. The mean ± SD of age was 18.6 ± 3.7 years in cases and 18.3 ± 3.6 years in controls. There was no significant difference between the patient and control groups regarding sex or age (P > 0.05). According to the GAGS score, 14 (32.55%) had mild acne, 23 (53.48%) had moderate acne, and six (13.95%) had severe acne (Table 1).

**Table 1:** The demographics and clinical characteristics of the study participants SD: standard deviation

Characteristics		Cases (n = 43)	Controls (n = 48)	P-value
Age	< 20 years	28 (65.11%)	32 (66.67%)	0.876
	≥ 20 years	15 (34.89%)	16 (33.33%)	
	Mean ± SD	18.6 ± 3.7	18.3 ± 3.6	0.953
Gender	Male	9 (20.94%)	14 (29.16%)	0.366
	Female	34 (79.06%)	34 (70.84%)	
Severity of acne	Mild	14 (32.55%)	-	-
	Moderate	23 (53.48%)	-	
	Severe	6 (13.95%)	-	

The association between the development of acne vulgaris and insulin resistance is shown in Table 2, which demonstrates that 35 (81.39%) of acne cases exceed the cut-off point of the C-peptide, which is significantly higher than the control group's 13 (27.11%) (P = 0.001). However, an insulin resistance definition based on the cut-off point of the TyG index estimates a low percentage of insulin resistance among acne cases compared to what C-peptide defines (67.44% vs. 81.39%). Still, this is significantly higher than the control percentage (18.75%) (P = 0.001).

**Table 2:** The association between insulin resistance and acne vulgaris * Insulin resistance is defined as either C-peptide > 1.89 ng/ml or TyG index ≥ 4.49 TyG: triglyceride-glucose index; IR: insulin resistance

Insulin Resistance *		Cases (n = 43)	Controls (n = 48)	P-value
C-peptide	IR	35 (81.39%)	13 (27.11%)	0.001
	No IR	8 (18.61%)	35 (72.91%)	
TyG index	IR	29 (67.44%)	9 (18.75%)	0.001
	No IR	14 (32.56%)	39 (81.25%)	

The means ± SD of the C-peptide and TyG index are shown in Table 3, which demonstrates a highly significant difference between the cases and controls for both the C-peptide and TyG index (P = 0.001).

**Table 3:** The levels of C-peptide and TyG index among cases and controls SD: standard deviation; TyG: triglyceride-glucose index

Insulin Resistance Biomarkers (mean ± SD)	Cases (n = 43)	Controls (n = 48)	P-value
C-peptide	2.84 ± 1.23	1.68 ± 0.19	0.001
TyG index	4.43 ± 0.17	3.38 ± 0.19	0.001

Regarding the association between insulin resistance biomarkers and the gender of cases, the results showed that there is no significant difference in C-peptide or TyG index levels among males and females (P > 0.05), despite slightly higher means for both biomarkers among males (3.01 ± 1.32 for C-peptide and 4.48 ± 0.18 for TyG index) compared to females (2.67 ± 1.15 for C-peptide and 4.38 ± 0.17 for TyG index) (Table 4).

**Table 4:** The association between gender of cases and insulin resistance biomarkers SD: standard deviation; TyG: triglyceride-glucose index

Insulin Resistance Biomarkers (mean ± SD)	Males (n = 9)	Females (n = 34)	P-value
C-peptide	3.01 ± 1.32	2.67 ± 1.15	0.448
TyG index	4.48 ± 0.18	4.38 ± 0.17	0.128

Furthermore, similar findings were obtained for the associations between the C-peptide, TyG index, and the age of patients, as they are not significant (P > 0.05), but there are slightly higher levels of these two biomarkers among those with ages of 20 years or more (3.05 ± 1.27 for the C-peptide and 4.44 ± 0.22 for the TyG index) compared to those younger than 20 years (2.63 ± 1.19 for the C-peptide and 4.42 ± 0.12 for the TyG index) (Table 5).

**Table 5:** The association between age of cases and insulin resistance biomarkers SD: standard deviation; TyG: triglyceride-glucose index

Insulin Resistance Biomarkers (mean ± SD)	Age < 20 years (n = 28)	Age ≥ 20 (n = 15)	P-value
C-peptide	3.05 ± 1.27	2.63 ± 1.19	0.377
TyG index	4.44 ± 0.22	4.42 ± 0.12	0.795

**Figure 1:**
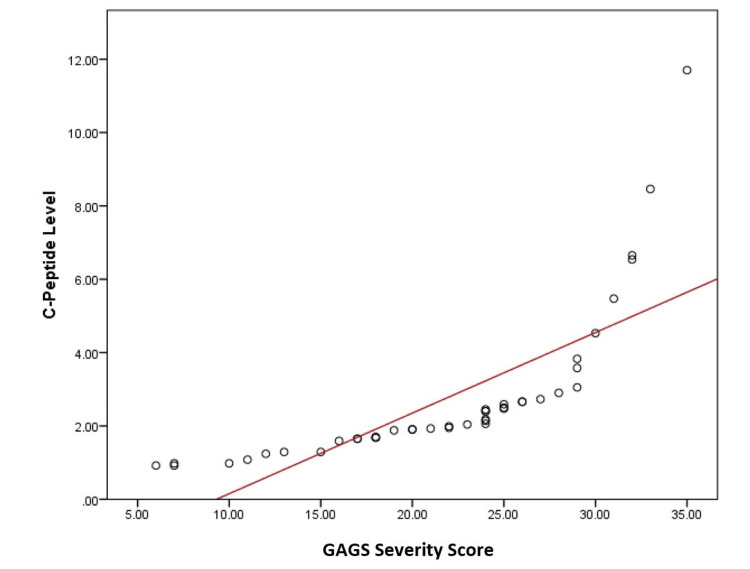
The correlation between C-peptide level and severity of acne GAGS: Global Acne Grading System

**Figure 2:**
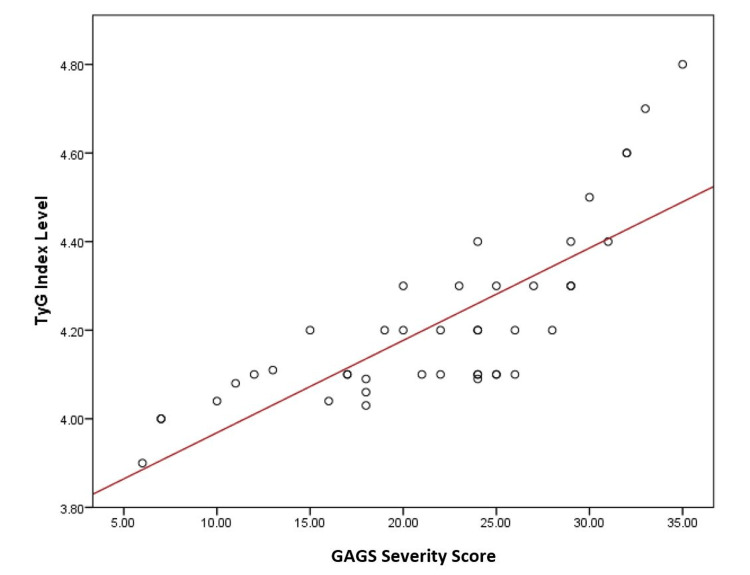
The correlation between TyG index level and severity of acne TyG: triglyceride-glucose index; GAGS: Global Acne Grading System

On the other hand, the association between insulin resistance biomarkers and severity of acne vulgaris clarified significant differences for both C-peptide and TyG index (P < 0.01) (Table 6). These associations are further confirmed by the correlation between C-peptide and TyG index levels and the severity of acne, which is shown in Figures 1 and Figure 2, demonstrating a strong and positive association between these biomarkers and increasing acne severity (r = 0.760 for C-peptide and 0.814 for TyG index) (P = 0.001).

**Table 6:** The association between acne severity and insulin resistance biomarkers SD: standard deviation; TyG: triglyceride-glucose index

Insulin Resistance Biomarkers (mean ± SD)	Mild (n = 14)	Moderate (n = 23)	Severe (n = 6)	P-value
C-peptide	1.33 ± 0.51	2.44 ± 0.32	4.75 ± 2.86	0.001
TyG index	4.12 ± 0.03	4.50 ± 0.17	4.68 ± 0.11	0.001

## Discussion

Insulin resistance has been found to be a strong predictor of metabolic illnesses in adults and the main component of metabolic syndrome. This has led to a new focus on research in this area. The condition is present when insulin levels are elevated relative to glucose levels. Thus, insulin resistance is linked to hyperinsulinemia by definition [10].

There is a scarcity of data regarding the association between hyperinsulinemia or insulin resistance and acne vulgaris; hence, this study aims to investigate further the existence of such an association.

There are many different methods to describe insulin sensitivity, such as the homeostasis model assessment for insulin resistance (HOMA-IR), C-peptide, and the TyG index. In the current study, we use the TyG index and C-peptide as markers for insulin sensitivity due to their availability in our locality and their cost-effectiveness characteristics.

C-peptide is a product of pro-insulin derived during insulin synthesis that roughly represents the amount of insulin produced and released. C-peptide is a biologically active molecule that can also be used as a diagnostic biomarker [11]. C-peptide is considered a powerful indicator of metabolic syndrome, emphasizing the importance of this biomolecule in metabolic syndrome diagnosis [12]. On the other hand, the logarithmized product of fasting triglycerides and fasting glucose levels (TyG) index is a surrogate for estimating insulin resistance compared to the HOMA-IR index because the insulin test is expensive and not available in most laboratories, especially in undeveloped countries [7].

The present study demonstrates that about 67.44 to 81.39% of the acne patients documented to have insulin resistance based on C-peptide and TyG index levels as well as the mean of these two biomarkers is significantly higher among cases with acne compared to control candidates, in addition to the positive and strong correlation between the level of these biomarkers and severity of acne and this is consistent with the findings of Emiroğlu et al. who observed a positive correlation between insulin resistance and severe acne vulgaris as well as a highly significant difference between the patient and control groups in terms of HOMA values [5]. Although the parameter they used is different, it yields the same conclusion, making the choice of the C-peptide and TyG index biomarkers of value in evaluating acne patients as it is well known that HOMA index calculation requires fasting insulin levels [13], which are more difficult to collect and not readily available in all laboratories compared to the C-peptide, triglyceride, and glucose levels required for the calculation of the TyG index. Another study done by Demir et al., who performed a case-control study on patients with insulin resistance and measured the C-peptide, triglyceride, and fasting glucose in addition to the HOMA index, also found that in patients with acne vulgaris there are increased levels of serum glucose and insulin, which contribute to insulin resistance [14]. A study done by Adebamowo et al. showed the same results and reported that hyperinsulinemia resulted in the flaring of acne [15]. Furthermore, a more recent systematic review and meta-analysis by Nickles et al. found that acne patients had a higher HOMA index, indicating greater insulin resistance, than controls (P = 0.01) [16]. According to the local literature about the relationship between metabolic disorders and acne vulgaris, a recent study conducted in the same center also showed a significant association and positive correlation between the development of acne and the severity of acne with the level of triglyceride and other lipid profile parameters, which reinforce the current research finding and link the possibility of metabolic derangement as a cause of acne and a risk factor for its severity [17].

On the other hand, a study done by Munichandrappa et al. did not find any significant correlation between acne and insulin resistance [18], which contradicts our results and many of the published studies; the reasons for that can be the small population size and the fact that the bulk of patients mainly belonged to the mild category of acne, while in our study, we took patients with moderate-to-severe acne vulgaris, as about two-thirds of our cases were moderate-to-severe.

In the current study, there is no significant association between gender, age, and insulin resistance biomarkers; however, we found a higher level of these biomarkers among post-adolescent males, and this is similar to the finding of a study conducted by Nagpal et al., who concluded that post-adolescent male patients with acne are more prone to have a higher prevalence of insulin resistance compared with the controls in their study [19], but this contradicts the findings of Kartal et al., who did a study on patients with acne and found a positive relationship between female acne and insulin resistance [20].

The findings of this study cannot be generalized to all acne patients since this was hospital-based research done at a single institution with a limited sample size. In addition, several diagnostic criteria are used to identify insulin resistance in the literature; however, due to a paucity of materials and high expenses, we rely only on C-peptide and the TyG index.

## Conclusions

We conclude that the majority of acne patients documented to have insulin resistance based on C-peptide and TyG index levels, as well as the mean of these two biomarkers, are significantly higher in acne cases compared to control candidates, in addition to the positive and strong correlation between the level of these biomarkers and the severity of acne. We recommend encouraging the control of dietary risk factors that increase insulin resistance and metabolic derangement during the acne treatment period, as well as encouraging physicians to look for these biomarkers when assessing acne severity and following up with the patient about these biomarkers throughout the treatment course. A future study looking for a link between a high-glycemic diet and the development of acne vulgaris is also recommended.
